# Eosinophil recruitment and activation in the central nervous system of patients with subarachnoid neurocysticercosis

**DOI:** 10.1186/s12974-025-03540-1

**Published:** 2025-09-26

**Authors:** Emily Miltenberger, Janitzio Guzmán, Rodaba Rahim, Miranda Yu, Michelle Makiya, Perla Adames Castillo, Soo Ching Lee, Theodore E. Nash, Amy D. Klion, Thomas B. Nutman, Elise M. O’Connell

**Affiliations:** Laboratory of Parasitic Diseases, National Institute of Allergy and Infectious Diseases, Division of Intramural Research, 10 Center Dr. Bldg 10 Rm 11N206, Bethesda, MD 20892 USA

**Keywords:** Cysticercosis, Neurocysticercosis, Eosinophilic meningitis, Helminth, Parasite, Eosinophils

## Abstract

**Background and objectives:**

Subarachnoid neurocysticercosis (SANCC) is the most severe manifestation of neurocysticercosis. Most complications (communicating hydrocephalus, ischemic stroke, aneurysm, and subarachnoid hemorrhage) are due to inflammation localized to the central nervous system (CNS). The role of eosinophils in the inflammation associated with SANCC has not been previously studied.

**Methods:**

Cryopreserved CSF collected as part of a clinical trial for neurocysticercosis (NCC) were assessed for analytes associated with eosinophil activation and recruitment using multiplex bead assays in both subjects with SANCC (*n* = 28) and in NCC-negative controls (*n* = 26). The SANCC patients underwent chart review for extraction of clinical variables as well as grouping by disease severity to identify analytes that may be associated with the development of more severe symptoms of SANCC.

**Results:**

Eosinophil granule proteins (EGPs – ECP, EDN, and EPO), markers of eosinophil activation, were elevated in the CSF of SANCC patients compared to controls. Moreover, the eosinophil-associated cytokines/chemokines IL-5, IL-13, IL-18, CCL24/eotaxin-2, and CCL26/eotaxin-3 were also significantly elevated in the CSF of SANCC patients compared to controls. In those for whom there were paired specimens (*n* = 13) from baseline and following cure, there was a significant reduction in these cytokines/chemokines (except CCL26/eotaxin-3). The percentage of CSF white blood cells that were eosinophils was positively correlated with EDN, EPO, IL-5, IL-13, CCL24, CCL26, CCL8, CCL13, and CCL5/RANTES, as well as the time it took to achieve biomarker cure. When SANCC patients were subdivided between those with severe disease and those with non-severe disease, the levels of eosinophil cationic protein (ECP), the CCR3 ligands (CCL7 and CCL5), CCL4, IL-18, and IL-1RA discriminated clearly between these 2 groups.

**Discussion:**

These data provide evidence for eosinophil recruitment and activation in the subarachnoid space in patients with SANCC, as well as for a potential role of eosinophils in driving inflammation-associated complications.

**Supplementary Information:**

The online version contains supplementary material available at 10.1186/s12974-025-03540-1.

## Introduction

Neurocysticercosis (NCC) is an infection of the central nervous system (CNS) caused by the larval stage (metacestodes or cysts) of *Taenia solium*. Infection may involve the brain parenchyma and/or extra-parenchymal locations (ventricles, subarachnoid space). Subarachnoid neurocysticercosis (SANCC), or racemose neurocysticercosis, occurs when cysts are found in the basilar cisterns, Sylvian fissures, interhemispheric space, or surrounding the spinal cord. SANCC is considered the most severe form of NCC due to the chronic and relapsing nature of the inflammation, which can last for decades due to the ability of this form of the parasite to proliferate much like a tumor [1]. Ultimately, it is the chronic inflammation that is believed to lead to the most common and severe consequences of SANCC, including communicating hydrocephalus, meningitis, ischemic stroke, aneurysms, and hemorrhagic complications [2, 3]. This inflammation is typically observed when the cyst(s) begin to degrade, either by natural involution or as a result of anthelmintic therapy with albendazole and/or praziquantel [2].

To reduce the inflammation provoked by anthelmintic therapy, patients with SANCC are typically treated concomitantly with immunosuppressive drugs, most commonly systemic corticosteroids, for weeks to months [4]. Long term treatment with corticosteroids can cause serious side effects, including osteoporosis, onset or worsening of diabetes, joint avascular necrosis, cataracts and adrenal insufficiency [5]. A better understanding of the specific drivers of inflammation in SANCC could lead to the use of more targeted therapies with improved side effect profiles.

Eosinophils are well-established players in the inflammatory response against helminth infections. They are found in the CNS in close proximity to the *T. solium* larvae, and some degree of eosinophil pleocytosis is typically found in the CSF of patients with SANCC [6–11]. Eosinophilic inflammation has also been demonstrated in porcine and murine models of the disease [12–14]. Although evidence points to eosinophils as potential drivers of pathogenic inflammation in SANCC, there has been minimal investigation into their role in SANCC-associated inflammation to date.

To explore the role of eosinophils in SANCC, we sought to characterize the CSF inflammatory milieu in infected patients at prior to anthelmintic therapy at the time of and how it changes at the point of cure in those same patients. Previously, we have detailed the relationship between cytokine and chemokine levels in the CSF and treatment success in SANCC [15]. The current study sought to understand the role played by eosinophils both in the pathogenesis of SANCC and in driving a more severe phenotype. Our data suggest that eosinophils contribute significantly to the inflammation observed in SANCC and may be a potential target for adjunctive immunosuppressive therapy.

## Materials and methods

### Sample collection and selection

Cerebrospinal fluid (CSF) was collected from patients with subarachnoid neurocysticercosis (SANCC) as part of the NIH IRB approved protocol *Natural History of Treated Neurocysticercosis and Long-Term Outcomes* (NCT00001205). Written informed consent was obtained for all participants. SANCC was diagnosed by either (1) MRI demonstrating the presence of cysts in the basilar cisterns or sylvian fissures of the brain or around the spinal cord and serologic positivity, or (2) MRI evidence of meningeal enhancement in these same subarachnoid spaced in the presence of a detectable *Taenia solium* antigen (TsAg) and/or DNA by qPCR in the CSF [16, 17]and in the absence of suspected or detected co-existing shunt infection (i.e. bacterial meningitis). Following collection by lumbar puncture (LP), CSF was centrifuged and supernatant was stored at − 80 °C. Selection criteria for this analysis included a SANCC diagnosis and available CSF sample drawn either at the pre-treatment (“baseline”) disease timepoint (preferentially prior to anthelmintic therapy, otherwise within 1 week of therapy start) or at the point of clinical cure (the point of stopping anthelmintics based on imaging, patient symptoms, and CSF inflammation). Since many samples had been collected on patients that were seen prior to the routine use of antigen and qPCR assays, the baseline and clinically cured time points for many were based on clinical grounds (as indicated by the term “clinical cure”). Those who had anthelmintics stopped upon clinical and imaging grounds and not biomarker negativity were considered clinically cured after annual imaging evaluation revealed no new disease after a minimum of 6 years of follow up. More recent patients had therapy guided by these biomarkers. Ultimately, all CSF samples used in this study underwent quantification of Taenia antigen and *T. solium* qPCR except in cases where limited sample volume made this impossible (Table 1). Since historically patients have been managed based on clinical and radiologic grounds, we thought it was important to describe the inflammatory milieu in these patients both as they were originally classified as well as with more current biomarker information.

**Table 1:** Baseline clinical and laboratory data

Subject ID	Severe (hydrocephalus, stroke, aneurysm) or non-severe (no complication)	CSF WBC (cells/µL)	CSF eosinophils (% WBC)	dexamethasone equivalent (mg/day)	TSOL13 (Cq mean)	TsG10 (ng/mL)
1	Non-severe	12	-	0	34.9	26.2
2	Non-severe	34	11	0	33.9	0
3	Non-severe	0	0	0	40	4.9
4	Non-severe	82	27.9	0	40	156.8
5	Non-severe	49	0	0	34.3	5017.9
6	Non-severe	92	15	0	27.8	324.4
7	Non-severe	8	0	12	33.11	958.9
8	Non-severe	12	3	0	-	147.2
9	Non-severe	16	-	24	40	21
10	Non-severe	16	0	0	40	126.1
11	Non-severe	4	0	0	40	86.9
12	Severe	41	6	0	34.3	9629
13	Severe	78	0.9	8	30.4	1448
14	Severe	34	0	0	29.5	31.3
15	Severe	22	0	0	30.3	66.7
16	Severe	34	26	0	23.5	100.3
17	Severe	112	-	0	24.3	420.4
18	Severe	33	11	0	24.2	21.7
19	Severe	278	32.4	0	30.1	25.2
20	Severe	61	0	0	-	-
21	Severe	123	9	1.5	25.8	2690.9
22	Severe	15	2.8	0	28.4	738.7
23	Severe	44	10.6	0	28.7	1132.7
24	Severe	18	0	0	28.7	63.8
25	Severe	149	15	4	26.3	286.1
26	Severe	-	-	0	40	0
27	Severe	4	0	6	33.5	22.2
28	Severe	425	0	0	26.5	3082.3

“-” denotes data which could not be obtained due to limited sample volume

CSF from a total of 28 patients with specimens available at baseline was assessed (27 prior to anthelmintic therapy, 1 within 1 week of therapy start). This patient cohort was comprised of 16 men and 12 women. The median age at the point of CSF draw was 37 years (15–77 years). Patients’ countries of origin and/or suspected regions of infection included Angola (1), Brazil (1), El Salvador (6), Guatemala (7), Honduras (6), and Mexico (7). Of the 28 patients with baseline CSF, 13 patients had reached clinical cure and had available CSF for paired assessment. An additional 6 unpaired patients also had CSF available from after clinical cure and are represented in supplementary assessments.

In place of healthy donor CSF, which was unavailable when the study was conducted, CSF from 26 NCC-negative control donors was used. This CSF was obtained from the NIH Clinical Center from anonymized patients already receiving lumbar puncture (LP) for other, unspecified conditions. All control specimens tested negative for the presence of TsAg and parasite DNA using diagnostic ELISA and qPCR assays [16, 17].

### Clinical and laboratory data collection

Chart review was conducted for all subjects to extract demographic data, relevant medication, history of complications, in addition to data produced by the NIH Clinical Center Department of Laboratory Medicine (DLM). Relevant medication history included anthelmintic dose (albendazole and/or praziquantel) and steroid dose (dexamethasone equivalent) at the time of LP. History of complication during the course of disease was extracted from patient medical records. Using the history of complications, patients were classified as having either severe or non-severe disease. Patients who experienced hydrocephalus, intracranial hemorrhage, aneurysm, or stroke were considered to have severe disease. Extracted DLM data included peripheral white blood cell and eosinophil counts from specimen drawn the same day as or within three months of the date of LP; CSF glucose, protein, white blood cell and eosinophil counts were also extracted. See Table 1 for medication history and DLM data.

### Measurement of taenia antigen and parasite DNA

TsAg in the CSF was measured using the TsG10-Biotinylated TsG10 ELISA as previously described [16] with a modification in the final detection steps further detailed in the supplementary materials. The concentration of TsAg in ng/mL was reported for each sample run in duplicate (Table 1).

The presence of *Taenia solium* DNA in the CSF was measured by a previously described qPCR assay [17]. DNA from 50 µL of CSF was extracted by QIAsymphony using the QIAsymphony DSP Virus/Pathogen Midi Kit (937055; Qiagen, Germantown, MD, USA). The assay was run following the protocol as described [17] with modifications described in the supplementary materials. The Ct mean was reported for each sample run in triplicate where a value below 40 is a positive result (Table 1).

### Measurement of eosinophil granule proteins

The concentration of three eosinophil granule proteins (EGP) – eosinophil cationic protein (ECP), eosinophil derived neurotoxin (EDN), and eosinophil peroxidase (EPO) – were measured in all CSF specimens using an in-house multiplex suspension array assay [18]. Following reduction and alkylation of CSF specimens, samples were run without further dilution. All specimens were measured in duplicate.

### Measurement of human cytokines, chemokines, and growth factors

A MILLIPLEX multiplex bead assay (MILLIPLEX Human Cytokine/Chemokine/Growth Factor Panel A 48 plex Premixed Magnetic Bead Panel [HYCTA-60 K-PX48]; Merck KGaA, Darmstadt, Germany) was used to measure CSF levels of sCD40L, EGF, eotaxin (CCL11), FGF-2, Flt-3 L, fractalkine (CX3CL1), G-CSF, GM-CSF, GROa (CXCL1), IFN⍺2, IFN-𝛄, IL-1⍺, IL-1β, IL-1RA, IL-2, IL-3, IL-4, IL-5, IL-6, IL-7, IL-8, IL-9, IL-10, IL-12(p40), IL-12(p70), IL-13, IL-15, IL-17 A, IL-17E/IL-25, IL-17 F, Il-18, IL-22, IL-27, IP-10 (CXCL10), MCP-1 (CCL2), MCP-3 (CCL7), MCSF (CSF1), MDC (CCL22), MIG (CXCL9), MIP-1⍺ (CCL3), MIP-1β (CCL4), PDGF-AA, PDGF-AB/BB, TGF ⍺, TNF ⍺, TNF β, VEGF-A. Another MILLIPLEX multiplex bead assay (MILLIPLEX Map Human Cytokine/Chemokine Magnetic Bead Panel II [HYCP2MAG-62 K]; Merck KGaA, Darmstadt, Germany) was customized and used to measure CCL24/eotaxin-2, CCL26/eotaxin-3, MCP-2 (CCL8), MCP-4 (CCL13), TSLP, and IL-33. Multiplex bead assays were run on the Luminex system. Established reliability of these commercial assay kits and limited sample volume meant that these analytes were measured in singlicate for all specimens.

RANTES (CCL5) was measured using a commercial ELISA kit (Human CCL5/RANTES DuoSet ELISA [DY278]; R&D Systems, Minneapolis, MN, USA). Limited sample volume required that samples be run in singlicate; only 26 of 28 baseline specimens and 17 of 26 control specimens could be assessed. All 13 patients with paired pre and post treatment specimens had both assessed.

### Statistical analysis

Two-tailed Mann-Whitney tests were used to compare measured analyte concentrations between baseline CSF and controls, patients with severe and non-severe disease, and CSF taken at clinical cure and controls. Wilcoxon pairs signed-rank tests were used to compare analyte concentrations between paired baseline and clinically cured specimens. Spearman r values were computed to determine the correlation between measured analyte concentrations and time to cure as well as the percent of eosinophils in the CSF of SANCC patients at baseline. P values for Mann-Whitney, Wilcoxon, and correlation analyses were considered statistically significant when *p* < 0.05. PCA analysis was performed using the prcomp function in R. The contribution of individual analytes to the top two PCs (PC1 and PC2) were assessed. This analysis was used to identify the analytes (among those listed in supplementary materials) that most contributed to the differences between patients with severe and non-severe SANCC.

## Results

### Increased levels of eosinophils in the CSF of SANCC patients

Eosinophils are rarely found in the CSF. Eosinophils are rarely found in the CSF. Of the 2424 SANCC patients whose CSF underwent cytological review at presentation, just over half had detectable eosinophils in the CSF (Table 1). Of the 13 patients with CSF from paired baseline and clinically cured timepoints, 11 had cytological differential data available; in 5 of the 6 patients who had detectable eosinophils at baseline, there was a marked reduction or complete disappearance of eosinophils at the time of clinical cure.

### Activation of eosinophils in the CSF of SANCC patients

**Fig. 1:**
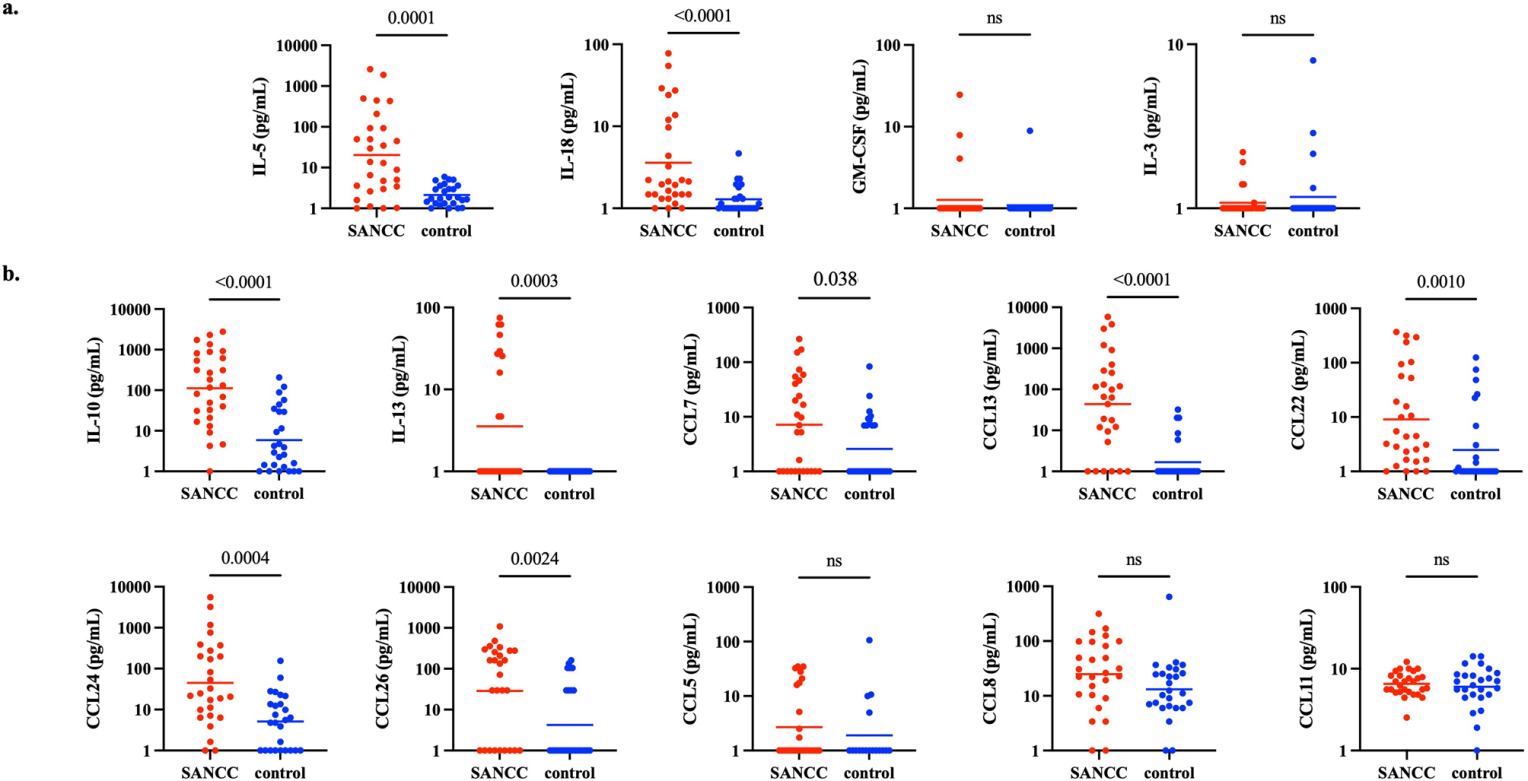
**a **Human cytokines and chemokines associated with eosinophilopoesis, (**b**) eosinophil recruitment and activation are significantly elevated in the CSF of patients with sANCC at baseline. With the exception of CCL5/RANTES, for 28 individual CSF specimens from SANCC patients and 26 individual CSF specimens from NCC-negative control subjects, analyte concentrations are reported in pg/mL (CCL5; baseline n=26, control n=17). Geometric mean is denoted by a horizontal line. P values are indicated on the graph where elevation is statistically significant (p<0.05) and otherwise marked as ns (not significant). Y-axes are long-10 scale; concentrations of all analytes are increased by one to accommodate the scale

Having demonstrated the presence of eosinophils in the CSF of those with SANCC, we next assessed their activation status by measuring the levels of the eosinophil granule proteins (EGPs: eosinophilic cationic protein, ECP; eosinophil derived neurotoxin, EDN; and eosinophil peroxidase, EPO) in the CSF SANCC patients (*n* = 28) and of NCC-negative controls (*n* = 26) as EGPs are the major degranulation products of activated eosinophils. EDN (*p* < 0.0001), EPO (*p* = 0.0029), and ECP (*p* = 0.0038) were significantly elevated in SANCC patient CSF (Fig. 1). Moreover, the concentrations of EDN (*p* = 0.0001, *r* = 0.65) and EPO (*p* = 0.004, *r* = 0.58) were significantly positively correlated with the percentage of eosinophils in the CSF (supplementary Fig. 1a).

### Cytokines and chemokines associated with eosinophilopoesis, eosinophil chemoattraction, and activation in the CSF of SANCC patients

**Fig. 2:**
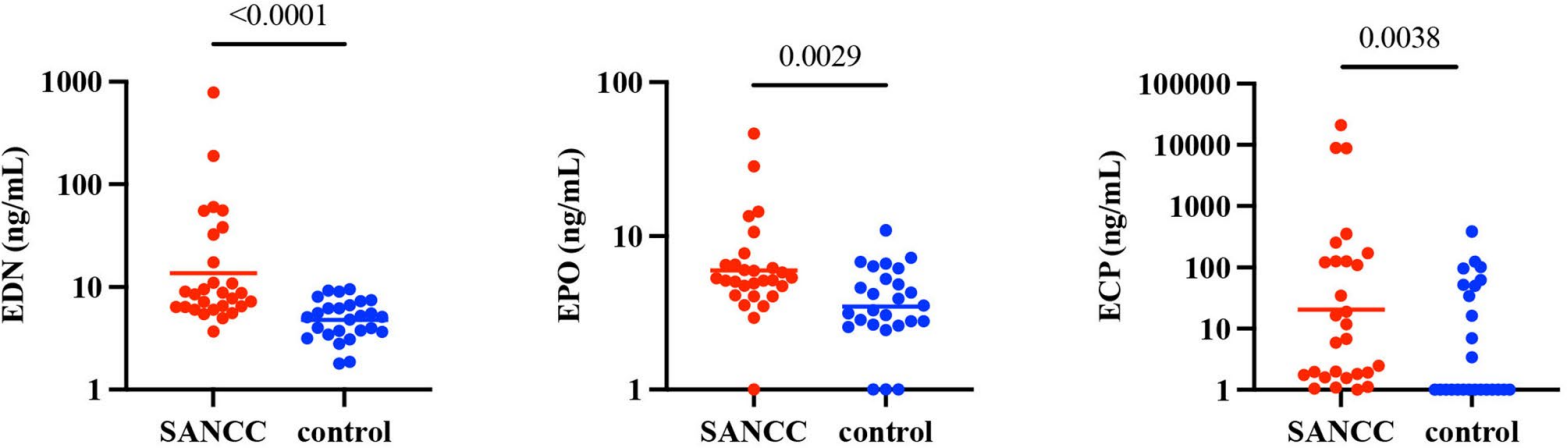
Eosinophil granule proteins are elevated in the CSF of patients with SANCC at baseline. Analyte concentrations are reported in ng/mL for 28 individual CSF specimens from SANCC patients and 26 individual CSF specimens from NCC-negative control subjects. Geometric mean is denoted by a horizontal line. *P* values are indicated on the graph where elevation is statistically significant (*p* < 0.05) and otherwise marked as ns (not significant). Y-axes are log-10 scale; concentrations of all analytes are increased by one to accommodate the scale

Using a multiplex bead assay, the concentrations of eosinophil-associated cytokines and chemokines were measured in the baseline CSF of 28 patients and in 26 NCC-negative CSF control specimens. Due to sample volume constraints, CCL5/RANTES was only measured in 26 SANCC patients and 17 of the controls. IL-5 and IL-18, cytokines involved in the differentiation of eosinophils from hematopoietic progenitor cells in the bone marrow [19–21]were elevated in SANCC patient CSF (*p* = 0.0001 and *p* < 0.0001, respectively; Fig. 2) compared to the negative control CSF. GM-CSF and IL-3, also involved in eosinophilopoesis, were not elevated (Fig. 2). Furthermore, CCR3 ligands (each known to be important in eosinophil trafficking), including CCL7 (*p* = 0.038), CCL13 (*p* < 0.0001), CCL24/eotaxin-2 (*p* = 0.0004), and CCL26/eotaxin-3 (*p* = 0.0024), were significantly elevated in SANCC patient CSF and there was a trend towards elevation of CCL8 (*p* = 0.055) (Fig. 1b). IL-13, which contributes to the induction of CCL24 and CCL26/eotaxin-3 by innate immune cells [22, 23] was elevated (*p* = 0.0003). CCL22, a chemokine that can be induced by IL-13 was also elevated (*p* = 0.001; Fig. 2). Interestingly, neither CCR3 ligands CCL5/RANTES nor CCL11/eotaxin-1, also understood to be potent eosinophil chemokines, were elevated (Fig. 2). Lastly, IL-10, a cytokine frequently reported as being elevated in the CSF of NCC patients [7, 15, 24]was also found to be elevated (*p* < 0.0001) in SANCC patients (Fig. 2).

Importantly, the concentrations of many of the cytokines and chemokines that were elevated at baseline in SANCC patient CSF had a strong and significant positive correlation with the percentage of eosinophils in the CSF (supplementary Fig. 1b). Notably, IL-5 (*r* = 0.71, *p* = 0.0002), IL-13 (*r* = 0.78, *p* < 0.0001), CCL8 (*r* = 0.52, *p* = 0.031), CCL13 (*r* = 0.75, *p* < 0.0001), CCL22 (*r* = 0.64, *p* = 0.0009), CCL24/eotaxin-2 (*r* = 0.71, *p* = 0.0002), and CCL26/eotaxin-3 (*r* = 0.60, *p* = 0.0032) demonstrated this correlation. Altogether, these results suggest that eosinophils are being recruited to the CNS in SANCC.

### Kinetics of eosinophil-associated cytokines and chemokines over the course of treatment

**Fig. 3:**
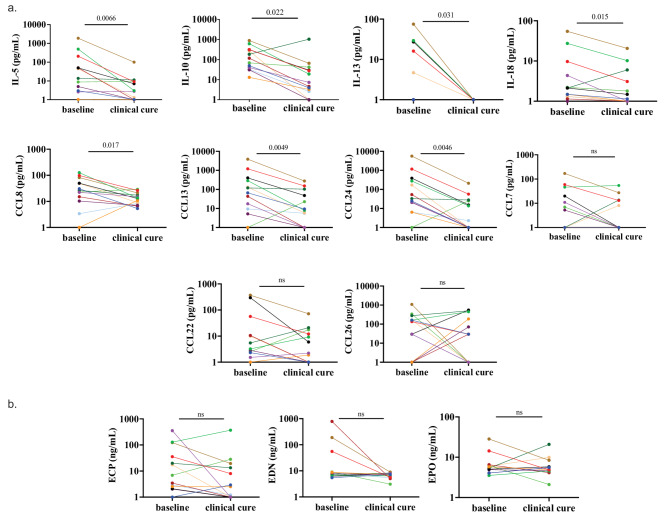
**a** Human cytokines and chemokines associated with eosinophilopoesis, eosinophil activation and recruitment significantly decrease in paired specimen at time of clinical cure; **b** whereas EGPs do not. Analyte concentrations are reported in (**a**) pg/mL or (**b**) ng/mL for CSF from 13 patients at baseline and clinically cured timepoints. Paired specimens share the same color in addition to being connected by a line. Colors are consistent between (**a**) and (**b**) for individual patients. *P* values are indicated on the graph where elevation is statistically significant (*p* < 0.05) and otherwise marked as ns (not significant). Y-axes are log-10 scale; concentrations of all analytes are increased by one to accommodate the scale

To assess what happens to the inflammatory milieu over the course of anthelmintic treatment, we measured eosinophil numbers, EGPs, and eosinophil-associated cytokines and chemokines in the CSF of 13 patients at the clinical cure timepoint for which we also had paired baseline CSF measurements. Of the cytokines and chemokines that were elevated compared to controls at the baseline timepoint, the concentrations of IL-5 (*p* = 0.0066), IL-10 (*p* = 0.022), IL-13 (*p* = 0.031), IL-18 (*p* = 0.015), CCL8 (*p* = 0.017), CCL13 (*p* = 0.0049), and CCL24/eotaxin-2 (*p* = 0.0046) all significantly decreased by the time of clinical cure; CCL7 and CCL26/eotaxin-3 did not decrease (Fig. 3a). An additional six unpaired clinically cured SANCC patient CSF samples were combined with the clinically cured cohort (total *n* = 19, supplementary table) and found to have elevated CCL13 (*p* = 0.0004) and CCL22 (*p* = 0.029) compared to NCC-negative controls (*n* = 26) (supplementary Fig. 2). EGPs were also measured across paired CSF samples from patients at baseline and following clinical cure; only EDN showed a trend towards a decrease (*p* = 0.077) (Fig. 3b).

### Increased cytokines and ECP in severe SANCC

Though our data suggest that eosinophils contribute to the inflammation observed in SANCC, it is unclear the extent of their involvement in the development of the severe complications that are thought to be a consequence of inflammation in some SANCC patients. To address this question, we categorized our 28 patients by disease severity. There were 17 patients who were classified as having severe (or complicated) disease, and 11 with non-severe (uncomplicated) disease (Table 1).

**Fig. 4:**
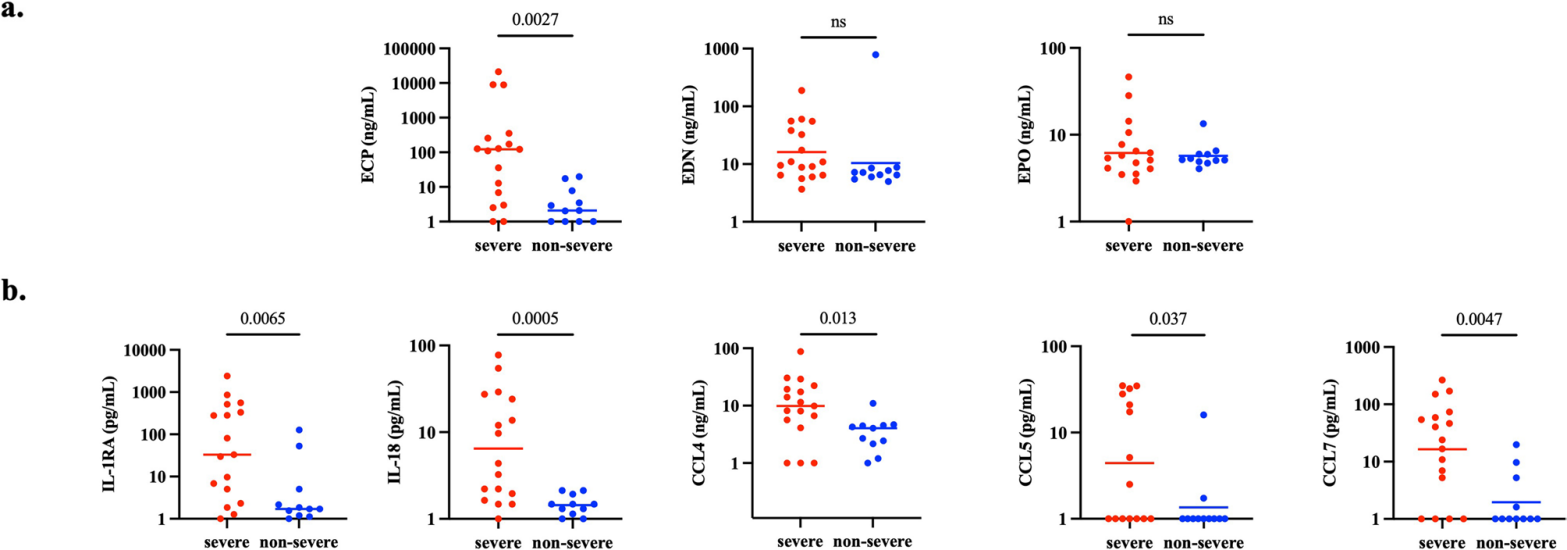
(**a**) ECP and (**b**) certain human cytokines and chemokines associated with eosinophilopoesis, eosinophil activation and recruitment are significantly elevated in the CSF of SANCC patients with severe disease at baseline. Analyte concentrations are reported in (**a**) ng/mL or (**b**) pg/mL for CSF from 28 patients with severe (*n* = 17) or non-severe (*n* = 11) disease. Geometric mean is denoted by a horizontal line. *P* values are indicated on the graph where elevation is statistically significant (*p* < 0.05) and otherwise marked as ns (not significant). Y-axes are log-10 scale; concentrations of all analytes are increased by one to accommodate the scale

**Fig. 5:**
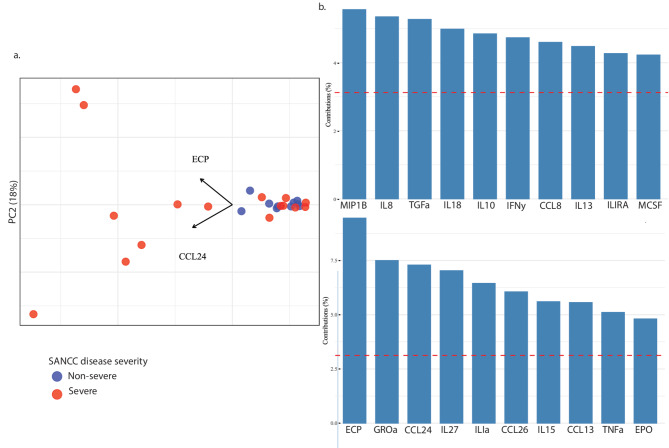
**a** PCA plot of severe and non-severe SANCC patients based on following analytes: TSOL13, TsG10, CCL11, FLT3l, GROa, IFNy, IL-1 A, IL-1RA, IL-4, IL-5, IL-6, IL-8, IL-10, IL-13, IL-15, IL-18, IL-27, CCL2, CCL7, MCSF, CCL22, CCL4, TGFa, TNFa, ECP, EDN, EPO, CCL24, CCL26, CCL8, and CCL13. Arrows indicate the top two analytes contributing to PC1 and PC2. **b** Contribution of top 10 analytes to PC1. **c** Contribution of top 10 analytes to PC2

In patients with severe disease, ECP was the only EGP that was significantly elevated (*p* = 0.0027) in the CSF compared to those with non-severe disease (Fig. 4a). Among those chemokines and cytokines that are associated with eosinophil signaling, IL-1RA (*p* = 0.0065), IL-18 (*p* = 0.0005), CCL4 (*p* = 0.013), CCL5 (*p* = 0.037), and CCL7 (*p* = 0.0047) were all significantly elevated in the CSF of patients with severe disease compared to those with non-severe SANCC (Fig. 4b). Principal component analysis (PCA) further revealed that ECP and CCL4 were the top analytes driving the separation of many severe patients from the predominantly non-severe group (Fig. 5).

### Relationship between duration of curative therapy and CSF levels of EGPs and CCR3

To begin to understand if the recruitment to and activation of eosinophils in the CNS has any relationship with the duration of anthelmintic therapy required to eradicate disease in SANCC patients, we conducted correlation analyses of the concentration of all measured analytes and continuous clinical variables with time to biomarker cure for the 23 patients who had reached cure at the time of the study. Biomarker cure was defined as CSF negativity for *T. solium* DNA (qPCR Ct of 40) and CSF antigen < 2 ng/mL. These criteria are currently used for declaring clinical cure in more recent patients, therefore patients seen before these criteria were established, and only considered clinically cured by means of radiologic and other clinical parameters, were excluded from this analysis (subject 3 and 7). There was a strong positive correlation between time to biomarker cure and the concentrations of ECP and EDN in baseline CSF (ECP, *p* = 0.002, *r* = 0.64; EDN, *p* = 0.002, *r* = 0.65) (supplementary Fig. 3). The concentrations of CCR3 ligands CCL26/eotaxin-3 (*p* < 0.0001, *r* = 0.80), CCL8 (*p* = 0.005, *r* = 0.60), and CCL13 (*p* = 0.015, *r* = 0.54), in SANCC patient CSF shared this strong positive correlation with time to biomarker cure (supplementary Fig. 3). CCL8 (*r* = 0.52, *p* = 0.008), CCL26/eotaxin-3 (*r* = 0.63, *p* = 0.001), ECP (*r* = 0.67, *p* < 0.0001), and EDN (*r* = 0.50, *p* = 0.012) likewise were positively correlated with the amount of parasite DNA present in the CSF (supplementary Fig. 3).

## Discussion

While eosinophils have been reported in the CSF of patients with extra-parenchymal disease [6–11]here we begin to explore the role of eosinophils in the inflammatory milieu of SANCC in humans. Persistent inflammation is responsible for the severe complications often experienced by patients with SANCC [3]. Thus, determining the role of eosinophils in the inflammatory milieu may open new avenues for targeted steroid-sparing agents. Here, we found that eosinophils, EGPs (markers of eosinophil activation), and cytokines and chemokines known to be associated with eosinophilopoesis and recruitment were elevated in the CSF of SANCC patients at the time of presentation compared to NCC-negative controls and the levels of these analytes markedly diminished at clinical cure.

EGPs are released when eosinophils degranulate during an immune response. Compared to NCC-negative controls, the concentrations of EDN, EPO, and ECP were elevated in SANCC patient CSF. EDN and EPO were also positively correlated with eosinophils in the CSF. As is seen in diseases driven by or involving eosinophilic inflammation, such as hypereosinophilic syndrome, eosinophilic esophagitis, and atopic dermatitis, elevation of EGPs in blood and body fluids provide an indirect measure of EGP deposition in tissue and can serve as a disease biomarker irrespective of peripheral blood eosinophilia [18, 25–31]. The elevations of EGPs in the CSF and correlation with CSF eosinophil counts is consistent with local eosinophil activation in the CNS of patients with SANCC. Many of the elevated cytokines and chemokines identified as eleveated at baseline significantly diminished between over the course of treatment by the time of clinical cure in the 13 paired samples. This diminishment of elevated cytokines and chemokines at clinical cure supports the hypothesis that eosinophils are being activated during SANCC, thus contributing to driving inflammation, and conditions at clinical cure more closely resemble the homeostatic condition.

In the baseline CSF SANCC patients, mediators of eosinophil differentiation from hematopoietic stem cells in the bone marrow – IL-5 and IL-18 (but not GM-CSF or IL-3) [19–21] – were elevated compared to NCC-negative controls. The marked elevation of IL-18 is particularly notable when considering recent work highlighting the role of IL-18 in promoting a pathogenic CD101 + CD274 + eosinophil phenotype associated with the pathogenesis of both eosinophilic esophagitis and asthma [19, 20, 32–34]. Elevated IL-18 in the brain and peripheral blood has also been associated with ischemic stroke and stroke induced inflammation [35] as well as with neurodegenerative disorders such as Alzheimer’s and frontotemporal dementia [36, 37]. Paired with work demonstrating the role of IL-18 in the development of pulmonary and hepatic fibrosis [38, 39] and the understanding that chronic meningitis leads to fibrosis of the meninges, IL-18 elevation in SANCC CSF may be relevant to the development of SANCC.

In addition to cytokines responsible for eosinophil differentiation, CCR3 ligands (CCL7, CCL13, CCL24/eotaxin-2, and CCL26/eotaxin-3) were also significantly elevated at baseline; whereas CCL11/eotaxin-1 and CCL5/RANTES, were not. IL-13, a cytokine involved in eosinophil recruitment through the promotion of CCL24/eotaxin-2 and CCL26/eotaxin-3 production by tissue cells at the site of inflammation [22, 40–42]was likewise elevated at baseline. Moreover, the concentration of many of these elevated analytes in the CSF had a strong positive correlation with the fraction of eosinophils present in the CSF. Together, these data suggest that eosinophil differentiation and recruitment to the site of inflammation are promoted in SANCC. Further, the pathway promoting this differentiation and subsequent recruitment appears to be distinct from some other eosinophil-mediated diseases that primarily rely on CCL11/eotaxin-1 and CCL5/RANTES and may instead operate through a CCL24 and CCL26 dominant pathway, potentially recruiting pathogenic eosinophils differentiated in the presence of IL-18.

To determine if any analytes, particularly those related to eosinophil recruitment and activation, might drive the development of more severe disease in SANCC patients, we grouped the 28 SANCC patients by severe (*n* = 17) and non-severe (*n* = 11) disease and compared their inflammatory profiles. We found that ECP, IL-18, IL-1RA, CCL4, CCL5, and CCL7 were elevated at baseline in severe SANCC patient CSF compared to those with non-severe disease. ECP, which belongs to the ribonuclease A superfamily, stands out among these elevated molecules not only because of its strong association with eosinophil activation but because of its pro-Th2 immunomodulatory capabilities and neurotoxic affects [43–46]. Combined with the elevation of IL-18 and the CCR3 ligands, CCL5 and CCL7, in patients with severe disease, these data suggest that more severe pathology in SANCC is driven, at least in part, by eosinophil-induced inflammation.

For the 23 patients for whom we had CSF at baseline and who had reached clinical cure, we looked at the relationship between the measured analytes and how long it took to achieve biomarker cure. We found significant positive correlations between the concentrations of all EGPs and the time to biomarker cure. Previously, our group found a strong correlation of time to clinical cure with the concentration of *T. solium* DNA [15]which we confirmed in this data set (presence of *T. solium* DNA reported under TSOL13 as Cq value in Table 1). Furthermore, we found that the concentrations of ECP and EDN, both similarly capable of immune-modulation and having neurotoxic potential [43, 45]were significantly correlated with time to biomarker cure, and inversely correlation with *T. solium* TSOL13 Cq amplification values, indicating increased degranulation products in the setting of increased parasite DNA. CCL26/eotaxin-3 and IL-18 were also strongly positively correlated with time to biomarker cure and with *T. solium* DNA concentrations. IL-18 has been described in pathologic eosinophil phenotype development and CCL26/eotaxin-3 in eosinophilic airway inflammation [42]. CCL26/eotaxin-3 has been associated with more severe asthma and has been shown to induce a stronger eosinophil migratory response [42, 47]. The positive correlation between these eosinophil-associated factors and time to biomarker cure suggests eosinophil-associated inflammation may impair an effective immune response, lengthening the time to elimination of parasite DNA and antigen.

There were several limitations to our study. Clinical parameters were incomplete for a small number of patients whose CSF either did not receive satisfactory analysis in the Department of Laboratory Medicine or for whom reduced sample volume made some tests impossible to conduct. Additionally, NCC-negative CSF from patients with other diseases was used in lieu of healthy control CSF. However, this limitation would likely underrepresent the degree of abnormalities in the cytokines and chemokines studied, as some of the control CSF samples may have had evidence of inflammation. Despite the lack of healthy control CSF, the use of NCC-negative CSF from patients at the NIH Clinical Center receiving LP for other reasons was able to provide insight into how the inflammation observed in SANCC differs from other CNS involved inflammatory diseases. This limitation was partially addressed through the analysis of paired samples which allowed us to observe how the inflammatory profile of SANCC patients changes before and after anthelmintic treatment, although the sample set was small. Nonetheless, we were able to demonstrate the activation of eosinophils in the CNS of patients with SANCC for the first time by showing elevated concentrations of EGPs in the CSF.

In sum, we have provided evidence that suggests the involvement of eosinophils in SANCC inflammation, and that eosinophil activation is associated with more severe pathology. Future work should focus on the role of eosinophils in the development of inflammatory complications of SANCC, and whether eosinophil depletion therapy may be an effective strategy for preventing the pathologic complications of inflammation in SANCC.

## Supplementary Information

Supplementary Material 1.

## Data Availability

No datasets were generated or analysed during the current study.
