# Supplementary material for: Eosinophil recruitment and activation in the central nervous system of patients with subarachnoid neurocysticercosis
Source: J Neuroinflammation. 2025 Sep 26;22:215. doi: 10.1186/s12974-025-03540-1 (PMC12465403; doi:10.1186/s12974-025-03540-1)
Supplement: Supplementary file 1 — Supplementary Material 1. [file 12974_2025_3540_MOESM1_ESM.docx]

**Supplementary Methods**

*Modifications to assays measuring Taenia antigen and parasite DNA*

The final detection steps of the *Taenia* antigen capture ELISA were modified to use 100 µL per well of a 1:10,000 dilution of alkaline phosphatase streptavidin incubated at 37°C for 1 hour (016-050-084; Jackson ImmunoResearch Laboratories, West Grove, PA, USA) followed after washing by 100 µL per well of 1 mg/mL of phosphatase substrate (S0942; Merck KGaA, Darmstadt, Germany) in sodium carbonate buffer (CUS-0242; KD Medical, Columbia, MD, USA) incubated at room temperature for 1 hour and 15 minutes. Plates were read at 405 nm.

The qPCR detecting *Taenia solium* DNA in the CSF used QuantiNova Probe PCR Master Mix and ROX Reference Dye provided in QuantiNova Probe PCR Kit (208252; Qiagen, Germantown, MD, USA) in place of Taqman reagents. 4 µL of extracted DNA or positive/negative controls were amplified in a total reaction volume of 10 µL. Plates were incubated at 95°C for 2 minutes followed by 2-step cycling between 95°C and 60°C for 5 seconds each for a total of 40 cycles.

**Supplementary Table. Clinical and Laboratory Data Including Additional Clinically Cured Patients**

“-” denotes data which could not be obtained due to limited sample volume

* specimen taken greater than 6 months after declared clinical cure date

**Supplementary Figures**


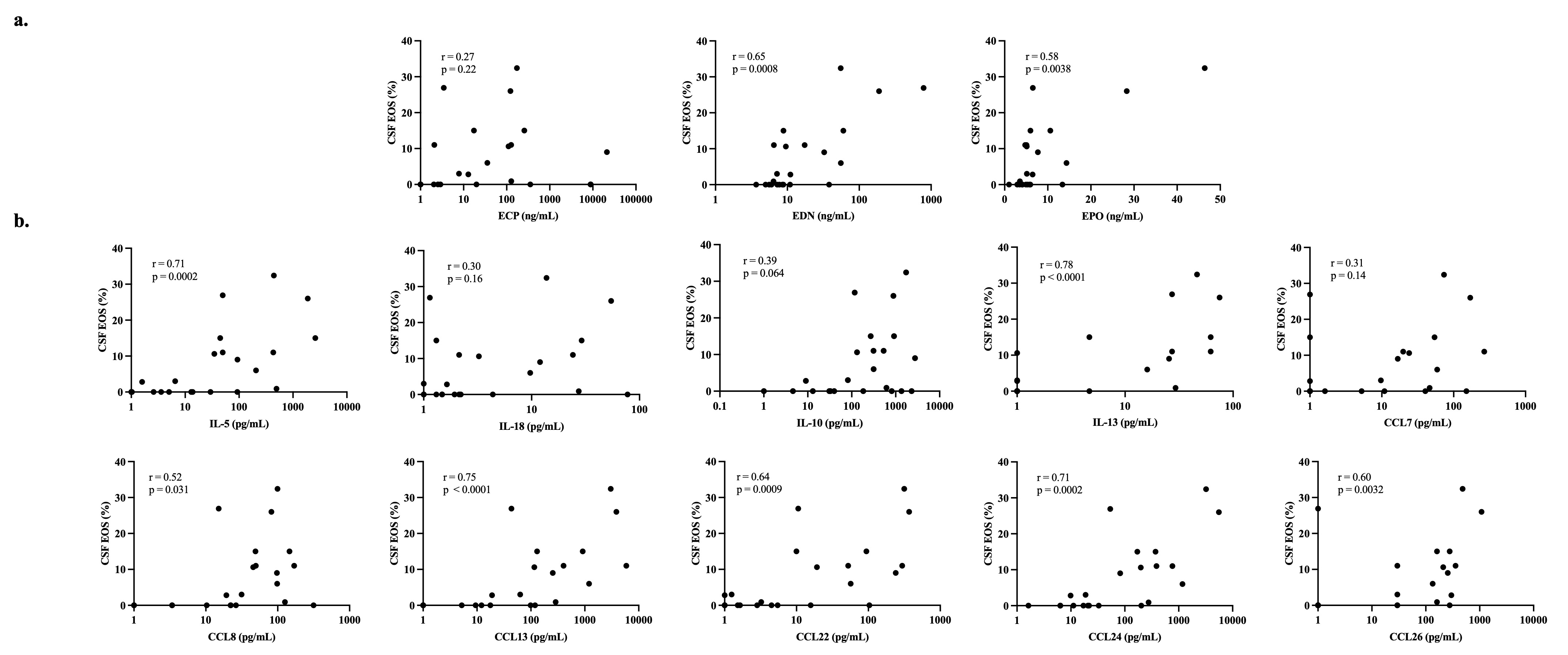


Supplementary Figure 1. (a) Concentrations of EGPs and (b) eosinophil associated cytokines and chemokines are correlated with the percent of eosinophils amongst WBCs in the CSF of patients with pre-treatment SANCC. Analyte concentrations are reported on the x-axis in (a) pg/mL or (b) ng/mL in the CSF from 28 patients; fraction of eosinophils of WBCs is reported as a percent on the y-axis. *P* and spearman R values are indicated on the graph; correlation is statistically significant when *p* < 0.05. X-axes are log-10 scale; concentrations of all analytes are increased by one to accommodate the scale.


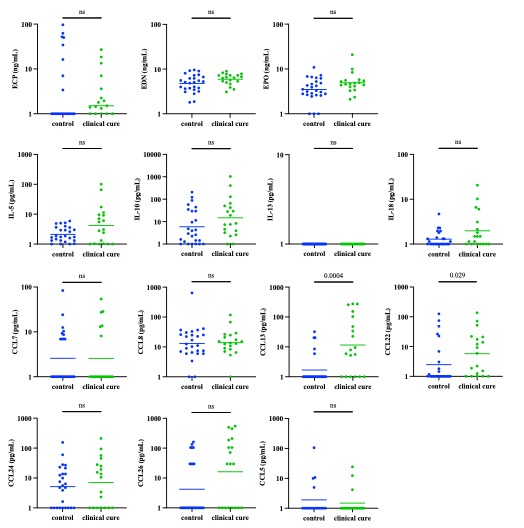


Supplementary Figure 2: Cytokines and chemokines in the CSF of SANCC at the time of clinical cure compared with unaffected control CSF.


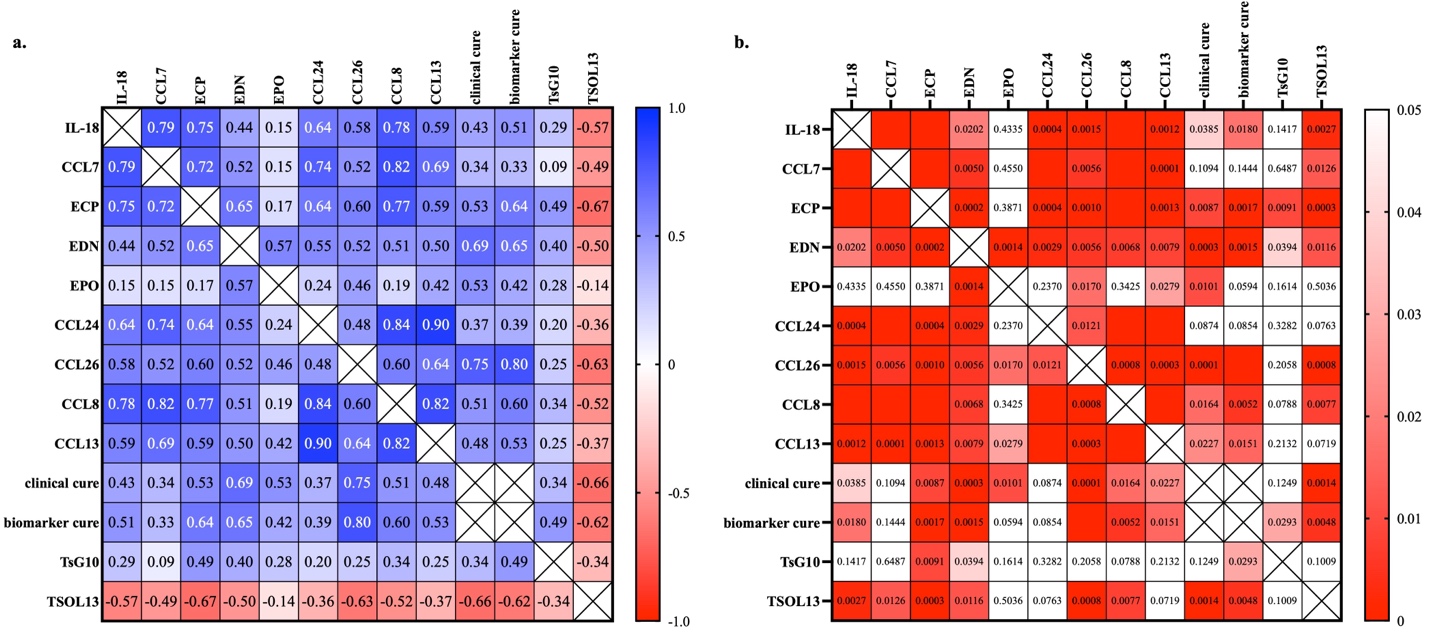


Supplementary Figure 3: Correlation matrices of eosinophil granule proteins with select chemokines and cytokines, T. solium CSF biomarkers, and cure. Panel a shows R values, panel b p values. TsAg, T. solium antigen; Tsol13, qPCR Tsol13.
